# OR7-001 – By chip pyrin binds the IRF2 promoter

**DOI:** 10.1186/1546-0096-11-S1-A102

**Published:** 2013-11-08

**Authors:** G Wood, Y Kanno, J Balow, H-W Sun, I Aksentijevich, DL Kastner

**Affiliations:** 1 IDS/MGB, NHGRI, Bethesda, MD, USA; 2 LCBS/MIIB, Bethesda, MD, USA; 3 Biodata Mining and Discovery Section/OST, NIAMS, Bethesda, MD, USA

## Introduction

The gene causing familial Mediterranean fever (FMF), *MEFV*, encodes a protein, pyrin, which is expressed at high levels in granulocytes, monocytes, dendritic cells and in some human myeloid leukemia cell lines, such as THP.1. Studies of pyrin localization show a cell-type dependency. In transfection experiments full-length pyrin is cytoplasmic and associates with the cytoskeleton. However, native pyrin is predominantly nuclear in granulocytes, dendritic cells, and synovial fibroblasts, but it is cytoplasmic in monocytes. Recent studies have implicated pyrin in the regulation of IL-1b and NF_Κ_B activation.

## Objectives

To provide additional molecular insight into pyrin function.

## Methods

RNA interference (RNAi) technique coupled with Affymetrix cDNA microarray analysis was employed to compare gene expression profiles between the human myeloid leukemia cell line, THP.1, expressing endogenous pyrin (scrambled control, SC) and cells in which the gene had been knocked down (si*MEFV*). Western blot and qRT-PCR analysis was used for validation. Promoter analysis was used to investigate the possibility of a common regulator among the subset of genes identified. Chromatin immuno-precipitation (CHiP) followed by quantitative PCR (qPCR) was used to validate binding.

## Results

We identified over 300 genes differentially expressed in si*MEFV* treated cells compared to (SC) with 1.4 fold difference and p < 0.05. Based on novelty and gene function, 10 down-regulated genes (*CD36*, *LY96*, *S100A8*, *CCR1*, *CD53*, *TIRAP*, *DEDD*, *SGK*, *MyD88*, *CD14*) were identified for further study. Using total RNA and protein from independent siRNA experiments, 7 of 10 genes showed a comparable mRNA and protein alteration consistent with microarray analysis. To investigate if the genes were being regulated by a common transcription factor, promoter analysis was used. We found that these genes showed enrichment of a binding site for interferon regulatory factors (IRFs, p=0.03). Additional screening of the 9 family members identified *IRF2* as the most significantly changed IRF transcription factor with a fold change of -3.4 and a p value of 0.008. Consistent with the computational analysis, si*MEFV* treated cells showed diminution in *IRF2* mRNA and protein compared to SC. Our previous studies identified *MEFV* as an interferon gamma (IFNγ) immediate early gene after IFNγ stimulation in monocytes. Thus, we decided to examine the possibility that pyrin could directly regulate *IRF2*. Using CHiP-qPCR to test this hypothesis, we demonstrated binding of pyrin within the promoter of *IRF2*.

## Conclusion

Our findings suggest that in THP1 cells pyrin might regulate expression of innate immune genes by binding to the promoter of the transcription factor *IRF2*. Further research is needed to examine the possibility that pyrin may bind to other DNA using CHiP coupled with next generation sequencing.

## Disclosure of interest

None declared.

